# Regulation of the Hepatocyte Cell Cycle: Signaling Pathways and Protein Kinases

**DOI:** 10.1155/2012/592354

**Published:** 2012-12-26

**Authors:** Pascal Loyer, Anne Corlu, Chantal Desdouets

**Affiliations:** ^1^Liver Metabolisms and Cancer, INSERM, UMR 991, Université de Rennes 1, CHU Pontchaillou, 35033 Rennes, France; ^2^Division of Cell Cycle, Regeneration and Liver Diseases, EMC Department, Institut Cochin, INSERM U1016, 24 rue du Faubourg St Jacques, 75014 Paris, France

The adult liver exhibits the remarkable ability to “regenerate” following surgical resection or toxic liver injuries. In normal liver restoration of hepatic tissue homeostasis occurs through rapid and partially synchronous proliferation of adult mature hepatocytes. The hepatocytes expressing the liver-specific functions responsible for the crucial hepatic metabolic pathways are quiescent cells that keep the ability to reenter the cell cycle. The fact that liver regeneration is supported by the active proliferation of highly differentiated hepatocytes rather than an expansion of progenitor cells is a unique situation among adult solid tissues. The hepatocytes, which exit quiescence and proliferate for a limited number of divisions present specific proliferation signaling pathways and a peculiar cell cycle regulation. In addition, polyploidy is another characteristic feature of mammalian adult hepatocytes that contributes to the specific molecular mechanisms underlying the cell cycle in hepatocytes. The entry into and progression through G1 phase of the cell cycle are orchestrated by complex networks of extracellular stimuli and intracellular signaling pathways inducing profound modifications of the gene expression required for the exit from quiescence and the cell cycle completion of the differentiated hepatocytes. Several lines of evidences also indicate that cell cycle regulators such as the Cyclin Dependent protein Kinases (CDKs) and their functional partners the cyclins and CDK inhibitors (CDKIs) show specific expression and/or activation patterns compared to the cell cycle in others cell types.

The special issue collected ten original research articles and reviews that present an update of various biochemical pathways underlying the cell cycle regulation of the adult hepatocytes. All the articles of this special issue explore in various extend the signaling pathways and the cell cycle protein kinases controlling the proliferation of adult mammalian hepatocytes. They also illustrate that many discoveries in this field benefited from the combined use of *in vivo* models of liver regeneration in rodents and *in vitro* models of both primary cultures of hepatocytes and in some extent established hepatoma cell lines. 

A first topic of interest of this special issue was the role of the liver microenvironment in the initiation of liver regeneration and more precisely the early stimuli leading to the reentry of the hepatocytes into the cell cycle also called “priming”. In that context, the release of cytokines and growth factors produced by nonparenchymal cells and the hepatocytes themselves, the early activations of downstream signaling pathways, signaling pathways, and the extracellular matrix remodeling were covered by several manuscripts. T. Nowatari et al. “*Regulation of signal transduction and role of platelets in liver regeneration*”, and in a less extent A. Corlu and P. Loyer “*Regulation of the G1/S transition in hepatocytes: involvement of the cyclin-dependent kinase cdk1 in the DNA replication*” provide a detailed update of the considerable literature published over the last 20 years that describes the productions of cytokines and growth factors by Kupffer and endothelial cells that orchestrate the proliferation of hepatocytes to counteract a loss of the liver mass. Notably, T. Nowatari et al. “*Regulation of signal transduction and role of platelets in liver regeneration*” focus on the more recently identified role of the platelets in liver regeneration. In an original article, F. Finot et al. “*Combined stimulation with the tumor necrosis factor alpha and the epidermal growth factor promotes the proliferation of hepatocytes in rat liver cultured slices*” provide the first description of hepatocyte proliferation in cultured rat liver slices. They further demonstrate in this *in vitro* cell system that the combined stimulation by proinflammatory cytokines such as the Tumor Necrosis Factor alpha (TNF*α*) and growth factor such the Epidermal Growth Factor (EGF) is required for the sequential activation of cell cycle regulators and the commitment to DNA replication. In their review, A. Corlu and P. Loyer “*Regulation of the G1/S transition in hepatocytes: involvement of the cyclin-dependent kinase Cdk1 in the DNA replication*” further detail the role of TNF*α*  in the extracellular matrix remodeling occurring in regenerating liver and cocultured rat hepatocytes prior any DNA replication. Conversely, the active proliferation of hepatocyte is greatly impaired during cirrhosis. V. C. Sanchez et al. “*Recovery of the cell cycle inhibition in *CCl_4_ *-induced cirrhosis by the adenosine derivative IFC-305*” report data showing a marked reduction of several cell cycle and mitochondrial regulators during CCl_4_-induced cirrhosis in rats. They further demonstrate that the cell cycle activity is partially restored by the adenosine derivate IFC-305 and suggest that the preservation of mitochondrial function may contribute to the recovery of proliferation in cirrhotic livers. 

The second and major theme of this issue is dedicated to the activation of intracellular signaling pathways and gene profile modifications controlling the priming of hepatocytes and progression in early G1 phase of the cell cycle. T. Garcin and T. Tordjmann “*Calcium signalling and liver regeneration*” reviewed the latest knowledge regarding the calcium signaling in liver regeneration following stimulation of the hepatocytes by calcium mobilizing agonists and activation of tyrosine kinase receptors, receptor channels, and G-protein-coupled receptors. This review emphasizes that calcium movement both in the cytoplasm and nucleus are clearly important events in the proliferative signaling through the transcriptional activation of immediate early genes and the control of the G1/S and G2/M transitions. Four other reviews develop distinct aspects of the signaling pathways especially phosphorylation events regulating the progression throughout the G1 phase and the commitment to DNA replication. The G1 phase of the cell cycle is *per se* under the control of extracellular growth factors activating a cascade of phosphorylation/dephosphorylation events that ultimately lead to the commitment to DNA replication. Beyond the G1/S transition, committed cells will proceed to DNA replication and mitosis regardless of the presence of growth factors in the extracellular microenvironment. The review by A. C. de l'Hortet and co-workers “*EGFR: A master piece in G1/S phase transition of liver regeneration*” reports in details the crucial role of the EGF receptors and its ligands in the G1/S transition and describes the activation of downstream phosphorylation events trigger by the EGFR. The authors also discuss the potential implication of this receptor in liver diseases including cancer but also metabolic disorders such as steatosis. Following stimulation by growth factors, it is now well established that the MAPK MEK/ERK pathway is crucial for both survival and proliferation in hepatocytes. J. P. Guégan et al. “*The MAPK MEK1/2-ERK1/2 pathway and its implication in hepatocyte cell cycle control*” provide an exhaustive overview of their own work and the literature regarding the role of this pathway in the hepatocyte cell cycle control. Furthermore, they emphasize the specific roles of ERK1 versus ERK2 both in normal and transformed hepatocytes. A. Gougelet and S. Colnot “*A complex interplay between Wnt/*β*-Catenin signalling and the cell cycle in the adult liver*” present in this issue another important pathway: the Wnt/*β*-catenin signaling pathway. Following the demonstration on the involvement of Wnt and its functional partner *β*-catenin in the liver carcinogenesis and the determination of the metabolic zonation in quiescent liver lobule, numerous publications have demonstrated the implication of the Wnt/*β*-catenin signaling pathway in the regulation of cell cycle genes such as the cyclin D1. Once again, two protein kinases play an important role in this pathway: the casein kinase 1 and glycogen synthase kinase 3. However, the authors have extended the discussion far beyond the induction of limited subset of genes by the activation of *β*-catenin and conclude with the unresolved dual role of this pathway in the balance between quiescence and proliferation in hepatocytes. Besides these canonical pathways, J. Pajaud et al. “*Regulation of signal transduction by glutathione transferases*” introduce the regulation of signal transduction by glutathione transferases (GSTs). The GSTs are well-known drug metabolizing enzymes but over the last decade a role of GSTs in the modulation of protein kinase activities has emerged. It is now well admitted that GSTs regulated protein kinases either by direct binding and/or by S-glutathionylation of catalytic subunits. In this article, the authors review the literature that link the GSTs to the regulation of apoptosis and cell cycle through the functional interactions with protein kinases such as c-jun terminal kinase (JNK) and the apoptosis signal-regulating kinase 1 (ASK1). 

A peculiar feature of the adult liver is the polyploidy of a large fraction of the hepatocytes. The polyploidization of the hepatocytes occurring during postnatal development is a noncanonical cell cycle that relies on an abnormal cytokinesis. G. Gentric et al. “*Hepatocytes polyploidization and cell cycle control in liver physiopathology*” review the molecular mechanisms and functional consequences of hepatocytes polyploidy during normal and pathological liver growth. They emphasize the role of insulin and the PI3K/Akt signaling pathway in the control of this process that they have largely contributed to decipher. 

All these contributions to our special issue deal with signaling pathways and protein kinases activating terminal executioner such as transcription factors and cyclin dependent kinases (cdks) and their regulatory subunits the cyclins. In a general review, A. Corlu and P. Loyer “*Regulation of the G1/S transition in hepatocytes: involvement of the cyclin-dependent kinase Cdk1 in the DNA replication*” summarize the latest discoveries on the cdks and cyclins involved in cell cycle control and discuss the emerging differences concerning the expression and regulation of the catalytic activity of cdk1 among the different mammalian cells. They also present the recent data demonstrating the involvement of cdk1 during the DNA replication in hepatocytes and the possible transcriptional control of the cdk1 gene by the TNF*α*  dependent signaling pathway. 

